# Successful management of severe diabetic ketoacidosis in a patient with type 2 diabetes with insulin allergy: a case report

**DOI:** 10.1186/s12902-019-0451-7

**Published:** 2019-11-11

**Authors:** Anh Dat Nguyen, Chinh Quoc Luong, Hieu Chi Chu, Van Khoa Dieu Nguyen, Chi Van Nguyen, Tuan Anh Nguyen, Quan Huu Nguyen, Ton Duy Mai, Dinh Van Nguyen, Bay Quang Nguyen, Thong Huu Tran, Phuong Viet Dao, Dat Tuan Nguyen, Nguyet Nhu Nguyen, Son Ngoc Do

**Affiliations:** 1 Department of Emergency and Critical Care Medicine, Hanoi Medical University, 01 Ton That Tung street, Kim Lien ward, Dong Da district, Hanoi, Vietnam; 2 Endocrinology and Diabetes Department, Bach Mai Hospital, 78 Giai Phong road, Phuong Mai ward, Dong Da district, Hanoi, Vietnam; 3 Allerology and Clinical Immunology Center, Bach Mai Hospital, 78 Giai Phong road, Phuong Mai ward, Dong Da district, Hanoi, Vietnam; 4 Department of Internal Medicine, Hanoi Medical University, Hanoi, Vietnam, 01 Ton That Tung street, Kim Lien ward, Dong Da district, Hanoi, Vietnam; 5 Emergency Department, Bach Mai Hospital, 78 Giai Phong road, Phuong Mai ward, Dong Da district, Hanoi, Vietnam; 6 Department of Allergy and Clinical Immunology, Hanoi Medical University, 01 Ton That Tung street, Kim Lien ward, Dong Da district, Hanoi, Vietnam

**Keywords:** Diabetic ketoacidosis, Type 2 diabetes, Insulin allergy, Recombinant human insulin, Continuous intravenous insulin infusion

## Abstract

**Background:**

Diabetic ketoacidosis (DKA) is an acute, major, life-threatening complication of diabetes that requires immediate treatment. Allergic reaction to insulin is rare, especially when using recombinant human insulin. The clinical presentation of insulin allergy can range from minor local symptoms to a severe generalized allergic reaction such as anaphylaxis. A limited number of cases have been reported on the treatment of severe DKA in patients with type 2 diabetes with insulin allergy. Here, we describe a patient with type 2 diabetes with insulin allergy in which severe DKA resolved after the initiation of continuous intravenous (IV) recombinant human insulin infusion.

**Case presentation:**

A 58-year-old man with type 2 diabetes initiated subcutaneous insulin administration (SIA) after failure of oral antidiabetic treatment. Symptoms of an allergic reaction developed, including pruritic wheals appearing within 10 min of injection and lasting over 24 h. Both skin prick and intradermal tests were positive with different types of insulin. Two days before admission, he stopped SIA because of allergic symptoms and then experienced weakness and upper abdominal pain. On admission, he was in severe metabolic acidosis with a pH of 6.984 and bicarbonate of 2.5 mmol/litre. The blood glucose level was 20.79 mmol/litre, BUN 4.01 mmol/litre, creatinine 128 μmol/litre, and urinary ketone 11.44 mmol/litre. Over 24 h, metabolic acidosis was refractory to IV fluids, bicarbonate and potassium replacement, as well as haemodialysis. Ultimately, he received continuous IV recombinant human insulin infusion at a rate of 0.1 units/kg/hour, in combination with haemodiafiltration, and no further allergic reactions were observed. On day 5, ketonaemia and metabolic acidosis completely resolved. He had transitioned from IV insulin infusion to SIA on day 14. He was discharged on day 21 with SIA treatment. Three months later, he had good glycaemic control but still had allergic symptoms at the insulin injection sites.

**Conclusions:**

In this patient, SIA caused an allergic reaction, in contrast to continuous IV insulin infusion for which allergic symptoms did not appear. Continuous IV recombinant human insulin infusion in combination with haemodiafiltration could be an option for the treatment of severe DKA in patients with diabetes with insulin allergy.

## Background

Diabetic ketoacidosis (DKA) is one of the most serious acute complications of diabetes that mainly occurs in patients with type 1 diabetes, but it is not uncommon in some patients with type 2 diabetes [1, 2]. The treatment for DKA includes correction of the fluid and electrolyte abnormalities and the administration of insulin. Moreover, patients with refractory DKA may improve following treatment with continuous venovenous haemodiafiltration (CVVHDF) and appropriate supportive care [3–5]. Allergic reaction to insulin is rare, especially when using recombinant human insulin, with a frequency of less than 1% in patients with diabetes [6]. The clinical presentation of insulin allergy can range from minor local symptoms to a severe generalized allergic reaction, specifically anaphylaxis [7, 8]. Insulin allergy can be managed safely and successfully by desensitization treatment [7, 9]. However, a limited number of cases have been reported on the treatment of severe DKA in patients with type 2 diabetes with insulin allergy. Here, we describe a patient with type 2 diabetes with an insulin allergy in which severe DKA resolved after the initiation of continuous intravenous (IV) recombinant human insulin infusion in combination with haemodiafiltration.

## Case presentation

**Fig. 1:**
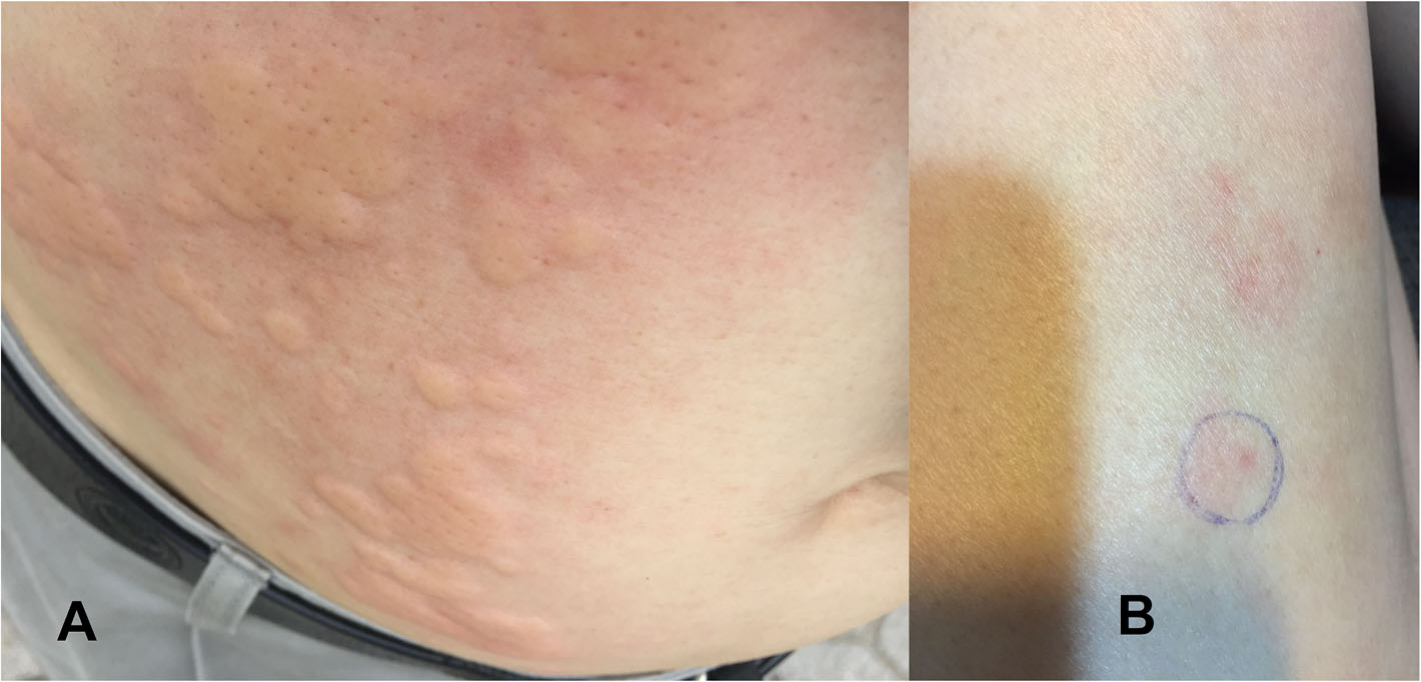
Allergic reactions to insulin. Before admission, the allergic reaction to insulin was characterized by urticaria with wheals (some of which are confluent) and flares (erythaema) on the abdominal wall surrounding the umbilicus (**a**). Over 3 months after being discharged, the local allergic reaction to insulin presents with an erythaema and swelling at the injection site – the outer side and front of the upper right thigh (**b**)

In August 2018, a 58-year-old man [height: 169 cm, body weight: 56 kg, and body mass index (BMI): 19.6 kg/m^2^] was admitted to our emergency department with upper abdominal pain, hyperglycaemia and metabolic acidosis. He had lived with type 2 diabetes for 16 years and had no history of any allergy, hypertension, hyperlipidaemia or renal diseases. Five months prior to admission, he initiated subcutaneous insulin administration (SIA) with the biphasic insulin analogue aspart after failure of sitagliptin and metformin therapies (HbA1c: 8.07% [65 mmol/mol]). Glycaemic control did not improve (HbA1c: 10.2% [88 mmol/mol]; total daily insulin dose was 20 UI), and aspart administration caused mild allergic symptoms. Aspart was then substituted by biphasic human insulin in which the total daily insulin dose increased up to 37 units. However, 5 months after the initiation of these regimens, he developed a pruritic wheal, especially distinct at the injection site (Fig. 1a). Pruritic wheals appeared within 10 min of injection and lasted over 24 h. The levels of fasting blood glucose and HbA1c deteriorated to 8.6 mmol/litre and 11.2% (99 mmol/mol), respectively. An allergy to insulin was then suspected. A skin prick test was carried out with different types of insulin [insulin aspart (NovoRapid®), recombinant human insulin (Actrapid® and Insulatard®), insulin glargine (Lantus Solostar®), and insulin lispro (Humalog®, Humalog mix®)] in which the test was positive for all of these types. Two days before admission, he stopped SIA because of an allergic reaction and was treated with anti-allergic drugs.

One day later, he experienced weakness and upper abdominal pain. On admission, clinical examination revealed a dehydrated patient with a heart rate (HR) of 130 beats/minute, a temperature of 37 °C and a systolic/diastolic blood pressure (BP) of 150/90 mmHg. He was tachypnoeic and dyspnoeic with a respiratory rate (RR) of 28 breaths/minute. He had hot and dry skin without pruritic wheals, isochoric pupils, and had no focal neurological deficit. He had normal breath sounds and a soft and non-tender abdomen. Electrocardiogram showed sinus tachycardia at a rate of 130 beats/minute. Echocardiography revealed normal chamber size and systolic function, without valvular lesions. Laboratory tests revealed high anion gap metabolic acidosis with an arterial blood pH of 6.984, bicarbonate of 2.5 mmol/litre and a serum anion gap (AG) of 26.4 mmol/litre. The arterial PO_2_ and PCO_2_ levels were 164.3 mmHg and 10.5 mmHg, respectively. Serum glucose was 20.79 mmol/litre, serum lactate was 1.5 mmol/litre, and urinary ketone was 11.44 mmol/litre. Serum potassium, sodium and chloride levels were 5.7 mmol/litre, 137.4 mmol/litre and 114.2 mmol/litre, respectively. Liver and renal function tests were normal, and there was a slightly elevated white blood cell count of 14.1 × 10^9^/l. He was admitted to our emergency ICU with a diagnosis of severe DKA in a patient with type 2 diabetes with an insulin allergy. Intravenous (IV) fluids, bicarbonate and potassium replacement and intermittent haemodialysis (IHD) were initiated. During the first 12 h, he received an initial 1 litre IV bolus of normal saline (0.9% NaCl) in the first hour, followed by a rate of 250 mL/hour, with 26 mmol of potassium chloride added per litre of normal saline. He also received 500 mL of sodium bicarbonate 1.4% solution over 2 h and then repeated as needed. However, his tachypnoea (35 breaths/minute) and metabolic acidosis persisted (arterial blood pH of 7.192, bicarbonate of 4.0 mmol/litre, PO_2_ of 156.1 mmHg, PCO_2_ of 10.3 mmHg, AG of 24.69 mmol/litre), prompting the initiation of CVVHDF using the Prismaflex® system (Gambro Lundia AB, Sweden) at the following settings: blood flow, 160 mL/minute; replacement volume, 1200 mL/hour; and dialysate, 1200 mL/hour. After 24 h of fluid resuscitation (6500 mL), he was haemodynamically stable and had 3500 mL of urinary output. However, he developed a decreased level of consciousness, agitation, and fatigue of his respiratory muscles. He was intubated for airway protection and was mechanically ventilated for respiratory support. Furthermore, hypotension (HR and BP were 120 beats/minute and 80/40 mmHg, respectively) occurred after intubation. A bolus of normal saline (1000 mL) was provided, and norepinephrine was administered at a rate of 0.3 μg/kg/minute. Haemodynamic stability was recovered after 1 h, with a HR of 110 beats/minute, BP of 120/60 mmHg, and measured CVP value of 8 cmH_2_O. Arterial blood gases revealed a worsening metabolic acidosis with an arterial blood pH of 7.022, bicarbonate of 2.5 mmol/litre and a serum AG of 25.75 mmol/litre. Renal function declined with a serum creatinine level of 198 μmol/litre. Serum glucose, potassium, sodium and chloride levels were 23.32 mmol/litre, 4.35 mmol/litre, 140.5 mmol/litre and 116.6 mmol/litre, respectively. CVVHDF and IV fluids and potassium replacement were continued. Although haemodynamic and respiratory stabilities were maintained, metabolic acidosis persisted. Further skin prick testing with different types of insulin [insulin aspart (NovoRapid®), recombinant human insulin (Actrapid®, Insulatard®, Mixtard®, Humulin R®, and Humulin N®), and insulin glargine (Lantus®)] only showed positivity to two (aspart, human) of these types. However, the intradermal test with these types was positive (the time of testing as shown in Additional file 1). A 40 mg dose of methylprednisolone sodium succinate and 10 mg of diphenhydramine were given in the event of the possible occurrence of a severe allergic reaction, and continuous IV infusion of recombinant human insulin was initiated at a rate of 0.1 units/kg/hour. Approximately 60 min after continuous IV infusion of insulin, he developed hypotension without any signs or symptoms of allergic reactions of the skin and mucosa, and the HR was 115 beats/minute and BP was 80/40 mmHg. Infusion of insulin was temporarily stopped followed by intravenous epinephrine administration at a starting rate of 0.15 μg/kg/minute in addition to an IV bolus of 1000 mL of normal saline. He regained haemodynamic stability after 30 min, including a HR of 110 beats/minute and a BP of 120/70 mmHg, and did not require any additional administration of epinephrine after 5 h. Continuous IV infusion of recombinant human insulin at a rate of 0.1 units/kg/hour continued without any events such as signs or symptoms of allergic reactions and hypotension.

On day 5 of follow-up, ketonaemia, metabolic acidosis (arterial blood pH of 7.465, bicarbonate of 18.4 mmol/litre and AG of 12.73 mmol/litre), and renal dysfunction (serum creatinine of 108 μmol/litre) had almost resolved, and CVVHDF was withdrawn. He did not require vasoconstrictors. Continuous IV infusion of recombinant human insulin continued and was adjusted according to blood glucose levels measured with a portable blood glucose meter. He was extubated on day 7 and transitioned from continuous IV insulin infusion to subcutaneous insulin (combined regular human insulin with insulin glargine) administration on day 14. He was discharged on day 21 with SIA (combined regular human insulin with insulin glargine) in combination with an oral antidiabetic drug (sitagliptin and metformin). Three months later, glycaemic control was gradually restored (HbA1c: 8.3% [67 mmol/mol]; total daily insulin dose was up to 44 UI); he still appeared to have mild allergic symptoms, such as local erythaema and swelling, especially distinct at the injection site of insulin glargine (Fig. 1b).

## Discussion and conclusions

DKA is not just the hallmark of absolute insulin deficiency in type 1 diabetes; it is increasingly being seen in people presenting with type 2 diabetes [2]. This condition is a complex disordered metabolic state characterized by hyperglycaemia, high anion gap metabolic acidosis, and ketonuria [10]. DKA must be distinguished from other causes of high anion gap metabolic acidosis, including lactic acidosis (which can rarely be associated with metformin), aspirin or acetaminophen toxicity and poisoning with methanol, ethylene glycol, and propylene glycol [10, 11]. The clinical and laboratory findings of our patient, however, revealed a typical DKA according to the diagnostic criteria proposed by the American Diabetes Association (ADA) [10]. DKA is an acute, major, life-threatening complication of diabetes that requires immediate treatment. Although DKA has a low rate of hospital mortality, the short-term risk of death is associated with recurrent DKA admissions in patients with diabetes [12]. However, this disorder can have significant mortality if misdiagnosed or mistreated, which is almost 100% without insulin therapy [13].

Over the first 30 h after admission, our patient was managed following the ADA guidelines for the treatment of hyperglycaemic crises in adult patients with diabetes [10], except for the administration of insulin. During this period of time, he also received IHD and CVVHDF for correcting severe DKA, but it persisted. The correction of metabolic acidosis with bicarbonate administration in the treatment of patients with DKA is controversial [14]. On rare occasions, IHD may be required to treat metabolic acidosis associated with renal failure. Combined CVVHDF with continuous IV insulin infusion was previously performed to treat successfully patients with refractory DKA [3, 5]. It is hypothesised that CVVHDF has a role in removal of plasma growth hormone (GH) and insulin growth factor 1 (IGF-1), similar to the clearance of other medium size molecules such as brain natriuretic peptide and procalcitonin [4]. In our patient, severe DKA was refractory to IHD, and duration of CVVHDF before initiation of continuous IV recombinant human insulin infusion was too short to draw conclusion dealing with clinical effect of CVVHDF in combination with other appropriate supportive care on the treatment of severe DKA. However, severe DKA in our patient resolved over a few days after starting combined CVVHDF with continuous IV recombinant human insulin infusion.

Immediate reactions to insulin preparations are believed to be immunoglobulin (Ig) E-mediated, type I immunologic reactions to insulin or to an additive [15]. The insulin-IgE complex binds to IgE receptors on the surface of basophils and mast cells, causing release of inflammatory mediators such as histamine, resulting in the minor local to severe generalized allergic reaction [16]. The IgE and IgG immunoassays were not available at our hospital; therefore, the patient was not assessed. However, his clinical features and skin tests showed a typical type-I allergic reaction according to the Gell and Coombs classification [17]. Anaphylaxis is the most severe presentation of an IgE-mediated drug reaction, and the skin and/or mucous membranes are involved in almost all cases [18]. In our patient, hypotension developed both before and after beginning continuous IV recombinant human insulin infusion, as well as after intubation without any signs or symptoms of allergic reactions of the skin and mucous membranes. However, his haemodynamic stabilities were rapidly obtained after adequate IV fluid replacement, and he did not require any further administration of epinephrine. Three risk factors for post-intubation hypotension were identified by multivariate analysis: decreasing mean arterial pressure pre-intubation, administration of neuromuscular blockers, and intubation complications [19]. In our patient, hypotension could therefore be attributed to decreasing mean arterial pressure pre-intubation and/or administration of neuromuscular blockers rather than severe allergic reactions.

Insulin allergy can be managed safely and successfully by desensitization treatment with the subcutaneous insulin route [7, 9, 20]. However, insulin tolerability in a severely insulin-allergic patient with diabetes could also be achieved by the use of intravenously injected insulin [21]. In this patient, treatment attempts of specific immunotherapy with subcutaneous administration of insulin, with continuous subcutaneous injection of insulin lispro, and with oral anti-allergic agents did not prevent frequent life-threatening allergic symptoms, especially after bolus injections with meals. Ultimately, no allergic reactions were observed after the authors applied the required insulin intravenously over a central line at a dose of 100 UI per 500 mL with a portable pump delivering 5–10 mL/hour, adjusted according to self-monitored blood glucose levels [21]. In our patient, severe DKA was refractory to IV fluid and sodium bicarbonate therapies and IHD. However, DKA resolved within several days after beginning continuous IV recombinant regular human insulin infusion in combination with CVVHDF, and no allergic symptoms were observed as previously described. Moreover, our patient appeared allergic symptoms with subcutaneous administration of insulin after transitioning from continuous IV insulin infusion. This phenomenon was also observed in the patient earlier, of whom the levels of anti-human insulin IgE although returned to normal, as did the levels of anti-human insulin IgG bound/total, without any adverse effect on glucose control, subcutaneous injection of regular insulin still caused immediate allergic reactions [21]. A previous study found that insulin treatment led to the production of antibodies against insulin [22]. A literature review had shown that the development of insulin antibodies was initially thought to be due to slight immunogenicity induced by the refining of preparations or the difference in amino acid sequences between species. When genetically engineered preparations of human insulin, however, are used, anti-human insulin IgG subclasses still are frequently detected in patients treated with insulin [23]. Thus, identical insulin molecules can behave in markedly different ways depending on the route of injection. Additionally, it is possible that the formation of anti-human insulin IgG is caused only by insulin molecules that are in contact with subcutaneous tissue [21]. These were assumed that some modification of insulin, such as aggregation, leads to the immunologic reactions [21, 24, 25].

In our patient, SIA caused an allergic reaction, in contrast to continuous IV insulin infusion, for which allergic symptoms did not appear. We believe that the presentation and progression of our patient indicated that continuous IV recombinant human insulin infusion in combination with haemodiafiltration could be an option for the treatment of severe DKA in patients with diabetes with insulin allergy.

## Supplementary information

**Additional file 1.** Timeline table for a case report

## Data Availability

Not applicable.

## Abbreviations

ADA: American Diabetes Association
AG: Anion gap
BP: Blood pressure
BUN: Blood urea nitrogen
CVP: Central venous pressure
CVVHDF: Continuous venovenous haemodiafiltration
DKA: Diabetic ketoacidosis
HR: Heart rate
ICU: Intensive care unit
Ig: Immunoglobulin
IHD: Intermittent hemodialysis
IV: Intravenous
PCO_2_: Partial pressure of carbon dioxide
PO_2_: Partial pressure of oxygen
RR: Respiratory rate
SIA: Subcutaneous insulin administration

## References

[1] Welch BJ, Zib I (2004). Case study: diabetic ketoacidosis in type 2 diabetes: “look under the sheets”. Clin Diab.

[2] Misra S, Oliver N, Dornhorst A (2013). Diabetic ketoacidosis: not always due to type 1 diabetes. Bmj.

[3] Lee SH, Kim BG, Cho AY, Kim SS, Shin HS, Kim JG (2015). A case of diabetic ketoacidosis with refractory metabolic acidosis successfully treated with continuous Hemodiafiltration. J Korean Soc Emerg Med.

[4] Mewawalla P, Jaiswal G, Moustakakis M, Sankaranarayanan N, Dasanu CA (2011). Refractory DKA as first presentation of acromegaly and a potential role for continuous venovenous hemofiltration in its successful management. Conn Med.

[5] Kawata H, Inui D, Ohto J, Miki T, Suzue A, Fukuta Y (2006). The use of continuous hemodiafiltration in a patient with diabetic ketoacidosis. J Anesth.

[6] Schernthaner G (1993). Immunogenicity and allergenic potential of animal and human insulins. Diabetes Care.

[7] Heinzerling L, Raile K, Rochlitz H, Zuberbier T, Worm M (2008). Insulin allergy: clinical manifestations and management strategies. Allergy..

[8] Hanauer L, Batson JM (1961). Anaphylactic shock following insulin injection: case report and review of the literature. Diabetes.

[9] Fujikawa T, Imbe H, Date M, Go Y, Kitaoka H (2012). Severe insulin allergy successfully treated with continuous subcutaneous insulin infusion. Diabetes Res Clin Pract.

[10] Kitabchi AE, Umpierrez GE, Miles JM, Fisher JN (2009). Hyperglycemic crises in adult patients with diabetes. Diabetes Care.

[11] Funes S, de Morais HA (2017). A quick reference on high anion gap metabolic acidosis. Vet Clin North Am Small Anim Pract.

[12] Gibb FW, Teoh WL, Graham J, Lockman KA (2016). Risk of death following admission to a UK hospital with diabetic ketoacidosis. Diabetologia..

[13] Kitabchi AE, Wall BM (1995). Diabetic ketoacidosis. Med Clin North Am.

[14] Kamel KS, Halperin ML (2015). Acid-base problems in diabetic ketoacidosis. N Engl J Med.

[15] Velcovsky HG, Federlin KF (1982). Insulin-specific IgG and IgE antibody response in type I diabetic subjects exclusively treated with human insulin (recombinant DNA). Diabetes Care.

[16] Castera V, Dutour-Meyer A, Koeppel M, Petitjean C, Darmon P (2005). Systemic allergy to human insulin and its rapid and long acting analogs: successful treatment by continuous subcutaneous insulin lispro infusion. Diabetes Metab.

[17] Coombs RRA, Gell PGH, Gell PGH, Coombs RRA, Lachman PJ (1975). Classification of allergic reactions responsible for clinical hypersensitivity and disease. Clinical aspects of immunology.

[18] Campbell RL, Hagan JB, Manivannan V, Decker WW, Kanthala AR, Bellolio MF (2012). Evaluation of National Institute of Allergy and Infectious Diseases/Food Allergy and Anaphylaxis Network criteria for the diagnosis of anaphylaxis in emergency department patients. J Allergy Clin Immunol.

[19] Smischney NJ, Demirci O, Diedrich DA, Barbara DW, Sandefur BJ, Trivedi S (2016). Incidence of and risk factors for post-intubation hypotension in the critically ill. Med Sci Monit.

[20] Pföhler C, Müller CSL, Hasselmann DO, Tilgen W (2008). Successful desensitization with human insulin in a patient with an insulin allergy and hypersensitivity to protamine: a case report. J Med Case Rep.

[21] Asai M, Yoshida M, Miura Y (2006). Immunologic tolerance to intravenously injected insulin. N Engl J Med.

[22] Berson SA, Yalow RS, Bauman A, Rothschild MA, Newerly K (1956). Insulin-I131 metabolism in human subjects: demonstration of insulin binding globulin in the circulation of insulin treated subjects. J Clin Invest.

[23] Fineberg SE, Kawabata TT, Finco-Kent D, Fountaine RJ, Finch GL, Krasner AS (2007). Immunological responses to exogenous insulin. Endocr Rev.

[24] Brange J, Andersen L, Laursen ED, Meyn G, Rasmussen E (1997). Toward understanding insulin fibrillation. J Pharm Sci.

[25] Maislos M, Mead PM, Gaynor DH, Robbins DC (1986). The source of the circulating aggregate of insulin in type I diabetic patients is therapeutic insulin. J Clin Invest.

