# Supplementary material for: Successful management of severe diabetic ketoacidosis in a patient with type 2 diabetes with insulin allergy: a case report
Source: BMC Endocr Disord. 2019 Nov 11;19:121. doi: 10.1186/s12902-019-0451-7 (PMC6849168; doi:10.1186/s12902-019-0451-7)
Supplement: Supplementary file 1 — Additional file 1. Timeline table for a case report [file 12902_2019_451_MOESM1_ESM.docx]

**Timeline table for a case report titled “Successful management of severe diabetic ketoacidosis in a type-2 diabetes patient with insulin allergy: a case report”**

| **Dates** | **Relevant Past Medical History and Interventions** | | |
| --- | --- | --- | --- |
| March 21, 2018  June 25, 2018  July 25, 2018  July 30, 2018 | The patient had lived with type 2 diabetes for sixteen years and had no history of any allergy, hypertension, hyperlipidaemia or renal diseases.  He initiated subcutaneous insulin administration (SIA) with the biphasic insulin analogue aspart after failure of sitagliptin and metformin therapies (HbA1c: 8.07% [65 mmol/mol]).  Glycaemic control did not improve (HbA1c: 10.2% [88 mmol/mol]; total daily insulin dose was 20 UI), and aspart administration caused mild allergic symptoms. Aspart was then substituted by biphasic human insulin in which the total daily insulin dose increased up to 37 units.  He developed a pruritic wheal, especially distinct at the injection site (Figure 1A). Pruritic wheals appeared within 10 minutes of injection and lasted over 24 hours. The levels of fasting blood glucose and HbA1c deteriorated to 8.6 mmol/litre and 11.2% (99 mmol/mol), respectively.  An allergy to insulin was then suspected. A skin prick test was carried out with different types of insulin [insulin aspart (NovoRapid®), recombinant human insulin (Actrapid® and Insulatard®), insulin glargine (Lantus Solostar®), and insulin lispro (Humalog®, Humalog mix®)] in which the test was positive for all of these types. Two days before admission, he stopped SIA because of an allergic reaction and was treated with anti-allergic drugs. One day later, he experienced weakness and upper abdominal pain. | | |
| **Date** | **Summaries from Initial and Follow-up Visits** | **Diagnostic Testing (including dates)** | **Interventions** |
| August 1, 2018 | One day later, he experienced weakness and upper abdominal pain. On admission, clinical examination revealed a dehydrated patient with a heart rate (HR) of 130 beats/minute, a temperature of 37°C and a systolic/diastolic blood pressure (BP) of 150/90 mmHg. He was tachypnoeic and dyspnoeic with a respiratory rate (RR) of 28 breaths/minute. He had hot and dry skin without pruritic wheals, isochoric pupils, and had no focal neurological deficit. He had normal breath sounds and a soft and non-tender abdomen. | Laboratory tests revealed high anion gap metabolic acidosis with an arterial blood pH of 6.984, bicarbonate of 2.5 mmol/litre and a serum anion gap (AG) of 26.4 mmol/litre. The arterial PO2 and PCO2 levels were 164.3 mmHg and 10.5 mmHg, respectively. Serum glucose was 20.79 mmol/litre, serum lactate was 1.5 mmol/litre, and urinary ketone was 11.44 mmol/litre. Serum potassium, sodium and chloride levels were 5.7 mmol/litre, 137.4 mmol/litre and 114.2 mmol/litre, respectively. Liver and renal function tests were normal, and there was a slightly elevated white blood cell count of 14.1 x 109/litre. | Intravenous (IV) fluids, bicarbonate and potassium replacement and intermittent haemodialysis (IHD) were initiated. During the first 12 hours, he received an initial one litre IV bolus of normal saline (0.9% NaCl) in the first hour, followed by a rate of 250 mL/hour, with 26 mmol of potassium chloride added per litre of normal saline. He also received 500 mL of sodium bicarbonate 1.4% solution over two hours and then repeated as needed. |
|  | After first 12 hours, his tachypnoea (35 breaths/minute) and metabolic acidosis persisted. | Arterial blood pH of 7.192, bicarbonate of 4.0 mmol/litre, PO2 of 156.1 mmHg, PCO2 of 10.3 mmHg, AG of 24.69 mmol/litre. | Initiation of CVVHDF using the Prismaflex® system |
| August 2, 2018 | After 24 hours of fluid resuscitation (6500 mL), he was haemodynamically stable and had 3500 mL of urinary output. However, he developed a decreased level of consciousness, agitation, and fatigue of his respiratory muscles. |  | He was intubated for airway protection and was mechanically ventilated for respiratory support. |
|  | Hypotension (HR and BP were 120 beats/minute and 80/40 mmHg, respectively) occurred after intubation. |  | A bolus of isotonic saline (1000 mL) was provided, and norepinephrine was administered at a rate of 0.3 µg/kg/minute. |
|  | Haemodynamic stability was recovered after one hour, with a HR of 110 beats/minute, BP of 120/60 mmHg, and measured CVP value of 8 cmH2O. | Arterial blood gases revealed a worsening metabolic acidosis with an arterial blood pH of 7.022, bicarbonate of 2.5 mmol/litre and a serum AG of 25.75 mmol/litre. Renal function declined with a serum creatinine level of 198 µmol/litre. Serum glucose, potassium, sodium and chloride levels were 23.32 mmol/litre, 4.35 mmol/litre, 140.5 mmol/litre and 116.6 mmol/litre, respectively. | CVVHDF and IV fluids and potassium replacement were continued |
|  | Although haemodynamic and respiratory stabilities were maintained, metabolic acidosis persisted. | August 15, 2018, further skin prick testing with different types of insulin [insulin aspart (NovoRapid®), recombinant human insulin (Actrapid®, Insulatard®, Mixtard®, Humulin R®, and Humulin N®), and insulin glargine (Lantus®)] only showed positivity to two (aspart, human) of these types. However, the intradermal test with these types was positive. | A 40 mg dose of methylprednisolone sodium succinate and 10 mg of diphenhydramine were given in the event of the possible occurrence of a severe allergic reaction, and continuous IV infusion of recombinant human insulin was initiated at a rate of 0.1 units/kg/hour. |
|  | Approximately 60 minutes after continuous IV infusion of insulin, he developed hypotension without any signs or symptoms of allergic reactions of the skin and mucosa, and the HR was 115 beats/minute and BP was 80/40 mmHg. |  | Infusion of insulin was temporarily stopped followed by intravenous epinephrine administration at a starting rate of 0.15 µg/kg/minute in addition to an IV bolus of 1000 mL of isotonic saline. |
|  | He regained haemodynamic stability after 30 minutes, including a HR of 110 beats/minute and a BP of 120/70 mmHg, and did not require any additional administration of epinephrine after 5 hours. |  | Continuous IV infusion of recombinant human insulin at a rate of 0.1 units/kg/hour continued. |
|  | Events (such as signs and symptoms of allergic reactions) and hypotension were not observed. |  |  |
| August 5, 2018 | On day 5 of follow-up, ketonaemia, metabolic acidosis (arterial blood pH of 7.465, bicarbonate of 18.4 mmol/litre and AG of 12.73 mmol/litre), and renal dysfunction (serum creatinine of 108 µmol/litre) had almost resolved. | Arterial blood pH of 7.465, bicarbonate of 18.4 mmol/litre and AG of 12.73 mmol/litre and serum creatinine of 108 µmol/litre. | CVVHDF was withdrawn. Continuous IV infusion of recombinant human insulin continued and was adjusted according to blood glucose levels measured with a portable blood glucose meter. |
| August 7, 2018 |  |  | He was extubated |
| August 14, 2018 |  |  | He was transitioned from continuous IV insulin infusion to subcutaneous insulin (combined regular human insulin with insulin glargine) administration. |
| August 21, 2018 | He was discharged |  | He was treated with SIA (combined regular human insulin with insulin glargine) in combination with an oral antidiabetic drug (sitagliptin and metformin). |
| December 27, 2018 | The patient’s glycaemic control was gradually restored; he still appeared to have mild allergic symptoms, such as local erythaema and swelling, especially distinct at the injection site of insulin glargine (Figure 1). | HbA1c: 8.3 % (67 mmol/mol) | Total daily insulin dose was up to 44 UI |
